# Multidimensional construct of life satisfaction in older adults in Korea: a six-year follow-up study

**DOI:** 10.1186/s12877-016-0369-0

**Published:** 2016-11-28

**Authors:** Hyun Ja Lim, Dae Kee Min, Lilian Thorpe, Chel Hee Lee

**Affiliations:** 1 Department of Community Health & Epidemiology, College of Medicine, University of Saskatchewan, 107 Wiggins Road, Saskatoon, SK S7N 5E5 Canada; 2 Department of Information Statistics, Duksung Women’s University, Seoul, Korea; 3 Clinical Research Support Unit, College of Medicine, University of Saskatchewan, Saskatoon, Canada

**Keywords:** Life satisfaction, Multidimensional, Older adults, Longitudinal study, Korean Retirement and Income Study (KReIS), GEE model

## Abstract

**Background:**

Aging raises wide-ranging issues within social, economic, welfare, and health care systems. Life satisfaction (LS) is regarded as an indicator of quality of life which, in turn, is associated with mortality and morbidity in older adults. The objective of this study was to identify the relevant predictors of life satisfaction and to investigate changes in a multidimensional construct of LS over time.

**Methods:**

This analysis utilized data from the large-scale, nationally representative Korean Retirement and Income Study (KReIS), a longitudinal survey conducted biennially from 2005 to 2011. Outcome measures were degree of satisfaction with health, economic status, housing, neighbor relationships, and family relationships. GEE models were used to investigate changes in satisfaction within each of the five domains.

**Results:**

Of a total 3531 individuals aged 65 or older, 2083 (59%) were women, and the mean age was 72 (s.d = ±6) years. The majority had a spouse (60.8%) and lived in a rural area (58%). Analysis showed that physical and mental health were consistently and significantly associated with satisfaction in each of the domains after adjusting for potential confounders. Living in a rural area and living with a spouse were related to satisfaction with economic, housing, family relationships, and neighbor relationships compared to living in urban areas and living without a spouse; the only outcome that did not show relationship to these predictors was health satisfaction. Female and rural residents reported greater economic satisfaction compared to male and urban residents. Living in an apartment was associated with 1.32 times greater odds of economic satisfaction compared to living in a detached house (95% CI: 1.14–1.53; *p* < 0.0001). Economic satisfaction was also 1.62 times more likely among individuals living with a spouse compared to single households (95% CI: 1.35–1.96; *p* < 0.0001). Financial stress index value was found to be a significant predictor of satisfaction with family relationships.

**Conclusions:**

Our study indicates that a single domain of LS or overall LS will miss many important aspects of LS as age-related LS is multi-faceted and complicated. While most studies focus on overall life satisfaction, considering life satisfaction as multidimensional is essential to gaining a complete picture.

## Background

In 2015, people aged 60 or over made up 12.3% (901 million) of the 7.3 billion global population, a proportion that is growing at a rate of 3.26% per year [1]. This number is projected to rise to 1.4 billion by 2030 and 2.1 billion by 2050. Compared to other nations, Asian countries such as Japan, China, and South Korea have been recognized to be aging more rapidly [1]. Global aging raises wide-ranging issues within social, economic, welfare, and health care systems which impact older adults and their families [2].

Life satisfaction (LS) is subjective well-being and is regarded as an indicator of quality of life. LS is influenced by individual demographic and clinical characteristics, as well as age [3–5]. Especially in the older population, LS should be considered as a multidimensional construct, including domains such as physical health, mental health, socio-economic status, social and family relationships, and the environment [6, 7]. As these domains are known to impact health, LS might be used to predict mortality and morbidity in older adults [8–10]. Since both LS and health are frequently thought to decline with age, LS is a popular outcome variable for evaluating older people’s lives and typically reflects broad domains in community-based and population-based studies of older adults [6, 11].

Although the assumption that LS declines in older age seems self-evident, particularly as health conditions deteriorate and living environments changes, research to date has been less definitive. Age- and sex-specific changes in LS among older adults remain unclear, and studies show inconsistent results. Some studies have found that age was positively correlated with LS [12–15], while other studies have detected a significant decline in LS over time [4, 16–19]. Still other studies have found stable levels of LS [20, 21]. Older women have been found by some to have lower levels of LS than older men [3, 22–24]. However, a few studies have also found that neither age nor gender was associated with LS [5, 25].

Physical and mental health have been significantly associated with LS in the older population [26–31]. Older adults who have retained their physical abilities and can perform activities of daily living tend to have higher LS, while those who perceive their health as poor tend to have lower LS. This mirrors much of the literature on depression in older adults, which suggests that those with serious medical illnesses, injuries, disability, isolation, and recent relocation appear to be more vulnerable to depression [32], whereas older adults in general, especially the younger ones, may have lower rates than young adults [33–36]. Depressive symptoms have been negatively correlated with LS in the older population, especially among older adults who live alone [26, 30, 37, 38]. Marital status, family status and household composition have also been associated with LS among older adults. Older adults who are living with their spouse, children, or in other types of cohabitation have been reported to have greater LS than those who are living alone [39–44]. These findings of poorer LS among the socially isolated older adults may stem from inadequate financial and emotional support, a lack of caregivers, or negative public perceptions that lead to poor mental health.

Financial security is an essential component of LS and is significantly associated with LS in the older population. Many studies suggest that financial difficulty in older individuals is related to depression and low LS [28, 31, 45, 46]. It is plausible that older adults with financial security have greater LS because they have financial resources to mitigate life’s challenges. However, a meta-analysis showed that the association between income and LS is relatively small, as quality of life in older people is not reduced by reduced income [47]. These individuals were found to be able to adjust their needs and desires to their financial situation.

Social support from friends and neighbors, as well as family, has also been significantly associated with the LS of older adults [23, 31, 42, 48–50]. Many studies have published findings that place of residence is associated with LS among the older population. Typically place of residence is often considered very broadly as either urban or rural. Living environment is relevant for older adults well-being and aging well, partly for enabling social engagement but studies show inconsistent results. Some studies show that urban residents have higher life satisfaction than rural residents [46, 51], while other studies were conducted in either rural or urban areas so comparisons were not possible [41, 44, 52]. Most studies examined place of residence in association with health, but very few studied LS. Huang found that a majority of older people in urban areas have a pension and enjoy other social welfare privileges and therefore, urban older adults have higher life satisfaction than rural older adults [53]. Millward [51] also found that life satisfaction varied significantly by urban–rural zones, including the inner city, suburbs, inner commuter belt, and outer commuter belt. In their study, older adults in the inner city had the highest LS [51]. Other studies have found the opposite results. Rural communities still gap behind in income distribution, access to affordable healthcare systems, social welfare programs and benefits and education [54]. However, older adults that lived in a rural environment presented a higher LS score than the ones living in urban settings because old adults living within a relatively steady social network, which provides regular contact over time, have high LS [55, 56]. Overall, literature related to LS in older adults is somewhat lacking. Most studies on LS in older adults are limited by their focus on a single aspect of LS. Additionally, many studies use cross-sectional designs, which offer little understanding of how LS changes over time. It is clear that consideration of LS as a multidimensional construct is essential to obtaining a complete picture of an individual’s state of LS. The aims of our study were to investigate changes in a multidimensional construct of life satisfaction (including satisfaction with physical health, mental health, economic, housing, family relationships, and neighbor relationships) among older adults and further elucidate relationships between each component of life satisfaction and relevant predictors using a longitudinal study. To address the study objectives, we analyzed data from the six-year follow-up Korean Retirement and Income Study (KReIS) using GEE models.

The conceptual framework for this study is derived from the previous concepts, life course perspective and socio-ecological models to explain life satisfaction in older adults. Our study adopts the theoretical framework by Cummins as its foundation [57, 58]. For the general population, Cummins has proposed the Comprehensive Quality of Life Scale based on both empirical and theoretical grounds, which has been found to be valid, reliable and sensitive. It specifies seven domains: material well-being, emotional well-being, health, productivity, intimacy, safety, and community. An individual’s well-being can be efficiently and comprehensively measured through these seven domains, which can be summed to yield a single measure of well-being. Since official productivity (i.e. job employment) is not relevant for the older population, only the remaining six domains were used. Emotional well-being can be assessed in part by evaluation of leisure activities, leisure time, or spiritual well-being.

**Fig. 1:**
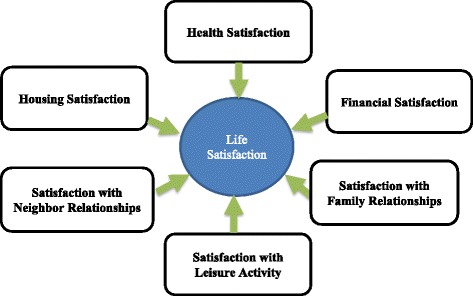
Multidimensional domains: revised conceptual model for life satisfaction in old population (Cummins, [58])

Such emotional well-being predicted increased psychological well-being and lower depressive symptoms [59, 60]. Therefore, helping older adults to maintain participation in informal leisure pursuits has important implications for promoting well-being in later life [60]. Our study adopted revised multidimensional domains of LS in old population from the Cummins’ conceptual model (Fig. 1). Unfortunately, in the data set our study was based on, indicators of emotional well-being, such as leisure activity, were not available. LS is a multidimensional construct in our study, with five satisfaction domains that include physical and mental health, economic, housing, family relationships, and neighbor relationships. To our knowledge, no previous study has assessed which factors are important predictors of LS change in older adults over time within each component of life satisfaction. Our primary hypothesis was that there are changes of LS in older adults. We also expected to find common but differing predictors among multidimensional LS domains. Thus, our second hypothesis was that demographic and environmental characteristics are predictors of each component of life satisfaction.

## Methods

### Data and sample

Korea is a country with a rapidly growing percentage of older adults and a relatively recently instituted national pension system (since 1988) which does not yet cover or is not enough for most older adults [61]. In 2014, the rate of poverty in the Korean adults of over 65 years old reached the highest level among 34 OECD countries, of 49.6% [62]. Due to forced early voluntary retirement, mean retirement ages are earlier than in Western countries, and as such, financial insecurity is likely to be a major contributor to life satisfaction. The data for this study comes from the Korean Retirement and Income Study (KReIS), a longitudinal survey conducted biennially from 2005 to 2011. The KReIS used a stratified sampling frame taken from the Korean Population and Housing Census in 2000. A total of 8567 individuals aged 50 or older participated in the initial survey in 2005. The core questions that the survey asked covered a wide-range of topics, including demographic aspects, economic status, housing, retirement, health status, and satisfaction with life. For our study, baseline responses from individuals aged 65 or older at the initial survey were examined, as were their subsequent responses for each wave that followed as long as answers to satisfaction items were provided. A total of 3531 individuals in the initial 2005 survey met our study criteria, with subjects at follow-up assessments numbering 3041, 2697, and 2330 at 2007, 2009, and 2011, respectively.

### Measures

Outcome measures in this study were satisfaction with health, economic status, housing, neighbor relationships, and family relationships. Satisfaction with each item was originally assessed on a 5-point scale that asked, “To what extent are you satisfied with the item below?”, evaluated on a scale ranging from 1 to 5 (very unsatisfactory = 1, unsatisfactory = 2, fair = 3, satisfactory = 4, very satisfactory =5). Satisfaction outcomes in this study were dichotomized, combining ‘very satisfactory’ or ‘satisfactory’ as ‘satisfactory’, and ‘very unsatisfactory’ or ‘unsatisfactory’ or ‘fair’ as ‘not satisfactory’.

In general, people in Korea are culturally hesitant to use the extreme answer and so the proportions of the extreme responses in our study were very small. Thus the 5-point LS was converted to binary outcome, Satisfactory vs Not-satisfactory, even though it can result in a loss of information regarding the original rating distributions. To investigate determinants of successful aging related to LS, Rowe & Kahn distinguished “usual” aging (non-pathologic but high risk) and “successful” aging (low risk and high function) [63]. In our study, the LS outcomes grouped ‘Very Satisfactory/Satisfactory’ is representing “successful aging”. Besides, other studies with the older population also dichotomized the same way as we did [42, 46].

Predictor variables were gender, age, education, presence of spouse, residential area, number of family members in the household, household composition type, housing type, current physical and mental health status, private health insurance, household income, and household expense. Age was recorded in years at the time of the baseline 2005 survey and was categorized as groups aged 65–69, 70–74, 75–79, 80 years and older. Sex was coded 0 = male and 1 = female. Information about education was coded as 0 = no education, 1 = elementary school (Grade 1–6), and 2 = middle school (Grade 7–9) or higher. Residential area was categorized into two areas by population size: urban (population ≥ 50,000) was coded as 0 and rural (population < 50,000) was coded as 1. Household composition type was categorized as 1 = living alone, 2 = living with a spouse, and 3 = mixed arrangements. Housing type was categorized as 1 = detached house, 2 = apartment, and 3 = other types. Current physical and mental health status were dichotomized as 1 = good or very good and 0 = very poor, poor or fair. Using household income and household expenses, household financial stress index (%) was calculated as

$$ \frac{\left(\mathrm{household}\kern0.75em \mathrm{income}\kern0.5em -\kern0.5em \mathrm{household}\kern0.75em \mathrm{expense}\right)}{\mathrm{household}\kern0.5em \mathrm{income}}\kern0.5em \times \kern0.5em 100. $$

Household financial index indicates levels of financial adequacy in a household. A positive value means financially good enough while a negative value means financial difficulty.

### Statistical analysis

Data were first analyzed to examine distributions and checked for outliers. Descriptive statistics were used to summarize the baseline characteristics of the study subjects. Student’s *t*-test and ANOVA were used for group comparison of continuous variables. For group comparison of categorical variables, the Chi-square test was used. Cross-sectional satisfaction outcomes were first analyzed by year, and the Cochran-Armitage test was then applied to assess trend in the proportion of respondents who were satisfied within each satisfaction outcome during the 6-year follow-up period. Correlation analysis between LS outcomes and covariates was also conducted. In addition to the univariate and multivariate analysis, a generalized estimating equations (GEE) model was used to adjust for repeated measurements among the study participants. The GEE model accounts for all available data points, such that respondents with incomplete data sets are not excluded from analysis under the assumption that missing are occurred at random [64].

Briefly for the GEE model, let y_ij_ is the jth outcome for the ith subject and x_i_ be the corresponding covariate vector. Then the GEE model can be written as g(E[y_ij_ |**x**_i_]) = **X**_i_ **β** where g(.) is a link function. For our binary outcome, let π _ij_ = E(y_ij_) be the expected probability of Satisfactory LS for subject i at the jth measurement. Then with logit link function, the GEE model is,

$$ \log \left(\frac{\pi_{ij}}{1-{\pi}_{ij}}\right)= \log \left(\frac{P\left({y}_{ij}=1\Big|{x}_i\right)}{P\left({y}_{ij}=0\Big|{x}_i\right)}\right)={\mathbf{X}}_{\mathrm{ij}}\boldsymbol{\upbeta} . $$

The GEE method is an efficient and flexible analytic technique to estimate model parameters, while controlling for the within-subject correlation in longitudinal data [64]. Using GEE method, the multiple outcome measurements of data are pooled so that LS outcome measurement from the previous time period can be controlled.

Univariate and multivariate logistic GEE models were developed using logit links; correlation between repeated assessments was examined prior to selecting the most appropriate correlation structure. The dependent variable in the model was the study participant’s satisfaction outcome (1 = satisfactory, 0 = not satisfactory). The GEE models included the covariates of sex, age, and education at the time of the 2005 survey as time-invariant while the other predictors were regarded as time-varying covariates. In the model building process, only significant predictors with *p* < 0.1 from the univariate GEE model were considered for the multivariate models. In the final models, interactions among the main predictor were also examined. For the GEE model goodness-of-fit, the QICu (Quasilikelihood under the Independence model Criterion) statistic was used [65]. Odds ratios (OR) and 95% confidence intervals (CI) were calculated. All reported *p*-values were 2-tailed, and *α* = 0.05 was set for statistical significance. All statistical analyses were carried out using SAS version 9.4 (SAS Institute, Cary, NC, USA).

## Results

In this study, a total of 2083 (59%) were women, and the mean age at the baseline was 72 (s.d = ±6) years (72.4 ± 6.2 years for women and 71.4 ± 5.6 years for men). Of the total study sample, the majority had a spouse (60.8%) and received no education or only elementary schooling (71%). In terms of household composition type, 40% were living with a spouse and 60% were either single or in a mixed arrangement living with others. About 58% of the study population lived in rural areas with a population under 50,000, and 59% lived in a detached house. Regarding economic status, very few had private health insurance (6.1%), and the mean household financial index value was -151 (s.d = 1780). Of the study sample, 2065 (58.5%) had a negative household financial index, i.e. household expenses exceed household income. Only small proportions of subjects had good physical and mental health status (18.3 and 28.9%, respectively). Table 1 further presents descriptive statistics of baseline characteristics. Table 2 shows the correaltion between five dimensions of life satisfaction, the overall life satisfaction, and the time-variant covariates.

**Table 1:** Baseline demographic characteristics of the study subjects. (*N* = 3531)

Covariate	Number of patients (%)
Sex	
Male	1448 (41%)
Female	2083 (59%)
Age	
65–69	1507 (42.7%)
70–74	987 (28.0%)
75–79	592 (16.8%)
≥80	445 (12.6%)
Spouse	
Yes	2147 (60.8%)
No	1384 (39.2%)
Education	
No education	1248 (35.4%)
Elementary School (Grade 1–6)	1257 (35.7%)
Middle school (Grade 7–9)	397 (11.3%)
High school (Grade 10–12)	405 (11.5%)
More than High school	217 (6.2%)
Residential area	
Urban	1497 (42.4%)
Rural	2034 (57.6%)
Housing type	
Detached House	2076 (58.8%)
Apartment	967 (27.4%)
Others	488 (13.8%)
Household composition type	
Single adult	628 (17.8%)
Couple	1402 (39.8%)
Others	1497 (42.4%)
Physical health	
Very poor	828 (23.5%)
Poor	1384 (39.3%)
Fair	663 (18.9%)
Good	591 (16.8%)
Very good	52 (1.5%)
Mental health	
Very poor	356 (10.1%)
Poor	996 (28.3%)
Fair	1150 (32.7%)
Good	919 (26.1%)
Very good	96 (2.7%)
Private health insurance	
Yes	215 (6.09%)
No	3316 (93.9%)
Financial stress index	
≥ 0%	1466 (51.7%)
−100% - 0	1056 (37.2%)
< -100%	1009 (11.0%)

**Table 2:** Correaltion between five dimensions of life satisfaction, the overall life satisfaction, and the time-variant covariates

	Health	Finance	Housing	Family relationships	Neighbor relationships	Overall^a^
Sex	−0.160*	−0.036*	−0.053*	−0.055*	−0.001	−0.083*
Age	−0.057*	0.0001	−0.030*	−0.098*	−0.133*	−0.094*
Education	0.198*	0.155*	0.107*	0.081*	−0.008*	0.149*

*p-value <0.0001

^a^The sum of the five dimensions of life satisfaction

**Fig. 2:**
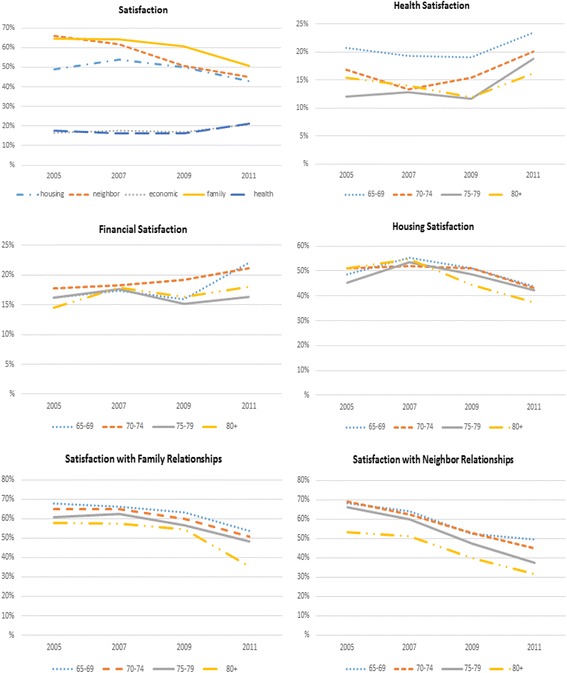
Proportion of subjects at each survey time point who reported being satisfied within each of the five satisfaction domains, all subjects combined and within each specific domain by age group. Note that values are the proportion of “Very Satisfied/Satisfied” responses from each LS dimension

**Fig. 3:**
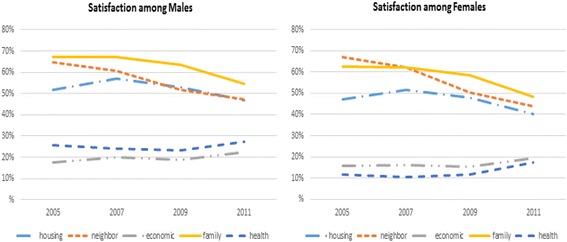
Proportion of subjects at each survey time point who reported being satisfied within each of the five satisfaction domains, stratified by sex. Note that values are the proportion of “Very Satisfied/Satisfied” responses from each LS dimension

**Fig. 4:**
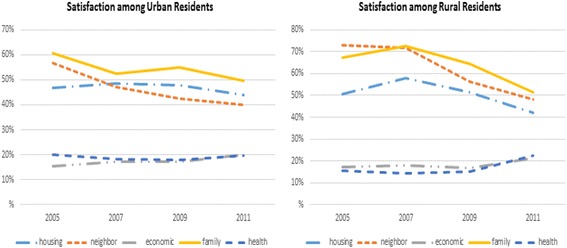
Proportion of subjects at each survey time point who reported being satisfied within each of the five satisfaction domains, stratified by residential area. Note that values are the proportion of “Very Satisfied/Satisfied” responses from each LS dimension

Descriptive statistics showed that satisfaction with family relationships, neighbor relationships, and housing ranged between 43 and 66% but health and economic status were small and relatively stable (Fig. 2). These temporal patterns were observed in both men and women and in both rural and urban areas. Except in regard to neighbor relationships, the proportion expressing satisfaction was consistently higher in men than women, especially in regard to health where the proportion was twice as high (Fig. 3). Comparing residential areas, rural participants were more frequently satisfied compared to urban participants except in the outcome of health satisfaction (Fig. 4). The data showed no differences in satisfaction among age groups regarding economic status, housing, and family relationships. However, subjects age 65–69 were more likely to be satisfied with their health, whereas those age 80 or older were less likely to be satisfied with neighbor relationships compared to the other age groups (Fig. 2). To further detail the associations between each satisfaction outcome and subject characteristics, results from the GEE models are presented.

### Health satisfaction

The GEE model showed that sex, presence of a spouse, education level, physical health status, and mental health status were significantly related to health satisfaction (Table 3). Aging was not associated with health satisfaction. Not living with a spouse resulted in a 25% reduction in odds of health satisfaction compared to living with a spouse (OR = 0.746; 95% CI: 0.634–0.891; *p* = 0.001). There was an interaction between sex and mental health (*p* = 0.0008). Men and women who reported good mental health were 2.71 and 4.29 times more likely to report satisfaction with their health compared to men and women with poor mental health, respectively (*p* < 0.0001). Among persons with good mental health, no difference between men and women was observed in health satisfaction (*p* = 0.933). However, among subjects with poor mental health status, women were less likely to be satisfied with their health compared to men (OR = 0.636; 95 CI: 0.503 – 0.804; *p* = 0.0002). Aging was not associated with health satisfaction.

**Table 3:** Health satisfaction. Estimation of odds ratio (OR), 95% confidence interval (C.I), and *p*-value from longitudinal random effects model

Covariate	Reference category	OR	95% CI	*p*-value
Sex	Female vs Male	0.801	0.671 – 0.957	0.015
Spouse	No vs Yes	0.746	0.634 – 0.891	0.001
Education	Elementary vs No education	1.014	0.841 – 1.223	0.883
	Middle or more vs No education	1.431	1.177 – 1.74	0.0003
Physical health	Good vs Poor	22.4	19.49 – 25.76	<0.0001
Sex by Mental health^a^	female/good vs male/good	1.009	0.818 – 1.245	0.933
	female/poor vs male/poor	0.636	0.503 – 0.804	0.0002
	female/good vs female/poor	4.293	3.52 – 5.235	<0.0001
	male/good vs male/poor	2.708	2.248 – 3.61	<0.0001
	female/good vs male/poor	2.732	2.197 – 3.398	<0.0001
	male/good vs female/poor	4.255	3.372 – 5.369	<0.0001

^a^The model has a significant interaction

### Economic satisfaction

Sex, age, education, residential area, housing, household composition type, physical health status, mental health status, and financial stress index were significantly associated with economic satisfaction (Table 4). Female and rural residents were more likely to report economic satisfaction compared to male and urban residents. Subjects living in an apartment were 1.32 times more likely to experience economic satisfaction compared to those living in a detached house (95% CI: 1.14–1.53; *p* < 0.0001). The coupled household was associated with 1.62 times greater odds of economic satisfaction compared to single households (95% CI: 1.35 –1.96; *p* < 0.0001). Good physical and mental health were significantly associated with economic satisfaction (*p* < 0.0001). Higher education and a positive financial stress index also showed higher economic satisfaction. There was an interaction between age and residential area (*p* = 0.0001). Comparisons of economic satisfaction between rural and urban residents were significant only in those age 65–69 (*p* < 0.0001), but not in the other age groups. Among urban residents, the older age groups were more likely to experience economic satisfaction. However, this trend was not shown among rural residents.

**Table 4:** Financial satisfaction. Estimation of odds ratio (OR), 95% confidence interval (C.I), and *p*-value from longitudinal random effects model

Covariate	Reference category	OR	95% CI	*p*-value
Sex	Female vs Male	1.406	1.201 – 1.647	<0.0001
Education	Elementary vs No education	1.139	0.958 – 1.353	0.141
	Middle or more vs No education	1.919	1.574 – 2.340	<0.0001
House type	Apartment vs Detached House	1.323	1.140 – 1.536	<0.0001
	Others vs Detached House	0.698	0.558 – 0.872	0.0002
Household composition	Couple vs Single	1.628	1.351 - 1.962	<0.0001
	Others vs Single	1.213	1.005 - 1.463	0.044
Physical health	Good vs Poor	1.747	1.514 – 2.016	<0.0001
Mental health	Good vs Poor	2.421	2.134 – 2.746	<0.0001
Financial stress	−100% - 0% vs ≥ 0%	0.652	0.576 – 0.739	<0.0001
	< -100% vs ≥ 0%	0.597	0.492 – 0.724	<0.0001
Age by Residential area^a^	rural/age 65–69 vs urban/age 65–69	1.705	1.390 – 2.093	<0.0001
	rural/age 70–74 vs urban/age 70–74	1.209	0.942 – 1.553	0.268
	rural/age 75–79 vs urban/age 75–79	0.763	0.540 – 1.078	0.071
	rural/age ≥ 80 vs urban/age ≥ 80	0.893	0.590 – 1.351	0.277
	urban/age 75–79 vs urban/age 65–69	1.975	1.452 – 2.686	<0.0001
	urban/age ≥ 80 vs urban/age 65–69	2.108	1.476 – 3.009	<0.0001
	rural/age 70–74 vs urban/age 65–69	1.868	1.485 – 2.350	<0.0001

^a^The model has a significant interaction; selected comparisons are presented

### Satisfaction with housing

Age, education, residential area, house type, household composition type, private insurance, physical health status, and mental health status were significantly associated with satisfaction with housing (Table 5). Rural residents were more likely to experience satisfaction with housing compared to urban residents (OR = 1.307; 95% CI: 1.184 -1.441; *p* < 0.0001). Having private insurance was also associated with a greater likelihood of experiencing satisfaction with housing compared to no private insurance (OR = 1.374; 95% CI: 1.152–1.639; *p* = 0.0004). There was no difference in housing satisfaction between males and females. Good physical and mental health were significantly associated with satisfaction with housing (*p* < 0.0001), as were increased age and higher education. Interaction between house type and household composition type was shown (*p* < 0.0001); single subjects or couples living in an apartment had greater odds of satisfaction with housing than those living in detached houses or other housing types.

**Table 5:** Housing satisfaction. Estimation of odds ratio (OR), 95% confidence interval (C.I), and *p*-value from longitudinal random effects model

Covariate	Reference category	OR	95% CI	*p*-value
Age	70 - 74 vs 65 - 69	1.092	0.979 – 1.218	0.114
	75 - 79 vs 65 - 69	1.109	0.968 – 1.272	0.136
	≥ 80 vs 65 – 69	1.241	1.048 – 1.468	0.012
Education	Elementary vs No education	1.062	0.948 – 1.188	0.30
	Middle or more vs No education	1.137	1.001 – 1.292	0.048
Residential area	Rural vs Urban	1.307	1.184 – 1.441	<0.0001
Private health insurance	Yes vs No	1.374	1.152 -1.639	0.0004
Physical health	Good vs Poor	1.538	1.368 – 1.728	<0.0001
Mental health	Good vs Poor	2.265	2.069 – 2.479	<0.0001
House type by composition^a^	APT/couple vs APT/single	0.986	0.777 – 1.253	0.91
	APT/couple vs Detach/single	3.033	2.516 – 3.655	<0.0001
	APT/single vs Others/single	2.812	2.002 – 3.95	<0.0001
	APT/single vs Detach/single	3.075	2.444 -3.869	<0.0001
	Detach/couple vs Detach/single	1.596	1.378 – 1.848	<0.0001
	Others/couple vs APT/single	0.371	0.278 – 0.495	<0.0001
	Others/couple vs Detach/single	1.14	0.895 – 1.453	0.289
	Others/single vs APT/single	0.445	0.356 – 0.555	<0.0001
	Others/single vs Detach/single	1.367	1.175 – 1.59	<0.0001

^a^The model has a significant interaction; selected comparisons are presented

### Satisfaction with family relationships

Sex, education, residential area, house type, household composition type, physical health status, mental health status, and financial stress index were significant factors in satisfaction with family relationships (Table 6). Female subjects and subjects who lived in an apartment were more likely to experience satisfaction in family relationships compared to male subjects and those living in detached houses (OR = 1.239; 95% CI: 1.111–1.338; *p* = 0.0001 and OR = 1.19; 95% CI: 1.063–1.333; *p* = 0.0026, respectively). Good physical and mental health were significantly associated with satisfaction with family relationships (*p* < 0.0001). Satisfaction with family relationships showed an interaction between residential area and household composition type (*p* < 0.0001). For singles living in rural areas, the odds of satisfaction with family relationships were higher than for singles living in urban areas (OR = 2.095; 95% CI: 1.813–2.421; *p* < 0.0001). However, satisfaction with family relations was not different between rural and urban areas for coupled and other household compositions. Aging was not a significant factor in satisfaction with family relations.

**Table 6:** Satisfaction with family relationships. Estimation of odds ratio (OR), 95% confidence interval (C.I), and *p*-value from longitudinal random effects model

Covariate	Reference category	OR	95% CI	*p*-value
Sex	Female vs Male	1.239	1.111 – 1.383	0.0001
Education	Elementary vs No education	1.306	1.166 – 1.463	<0.0001
	Middle or more vs No education	1.288	1.121 – 1.479	0.0003
House type	Apartment vs Detached	1.190	1.063 – 1.333	0.0026
	Others vs Detached	0.721	0.629 – 0.827	<0.0001
Physical health	Good vs Poor	1.533	1.34 - 1.755	<0.0001
Mental health	Good vs Poor	3.066	2.764 – 3.40	<0.0001
Financial stress	−100% - 0% vs ≥ 0%	0.883	0.798 – 0.976	0.015
	< -100% vs ≥ 0%	0.734	0.639 – 0.844	<0.0001
Residential area by Household composition^a^	rural/single vs urban/single	2.095	1.813 – 2.421	<0.0001
	rural/couple vs urban/couple	1.063	0.878 – 1.286	0.41
	rural/others vs urban/others	1.164	0.894 – 1.516	0.068
	rural/couple vs urban/single	1.762	1.468 – 2.114	<0.0001
	rural/couple vs urban/others	1.745	1.397 – 2.179	<0.0001
	rural/single vs urban/others	2.075	1.713 – 2.514	<0.0001

^a^The model has a significant interaction; selected comparisons are presented

### Satisfaction with neighbor relationships

Sex, age, residential area, housing type, household composition type, physical health status, and mental health status were significant factors in satisfaction with neighbor relationships (Table 7). Rural residents had odds of satisfaction with their neighbor relationships that was 1.729 (95% CI: 1.576–1.895; *p* < 0.0001) higher compared to urban residents. Subjects living in an apartment or other types of housing were significantly less likely to experience satisfaction with neighbor relationships compared to those living in detached houses (*p* < 0.0001). Good physical and mental health were significantly associated with satisfaction with neighbor relationships (*p* < 0.0001). However, individuals age 80 or older were significantly less likely to indicate satisfaction with neighbor relationships compared to the other age groups. Satisfaction with neighbor relationship also showed an interaction between sex and household composition type (*p* = 0.002). Among both couples and singles, females were 1.22 and 1.873 times more likely to be satisfied with neighbor relationships compared to males (*p* = 0.052 and *p* < 0.0001, respectively), but not for other types of household composition (*p* = 0.121).

**Table 7:** Satisfaction with Neighbor Relationships. Estimation of odds ratio (OR), 95% confidence interval (C.I), and *p*-value from longitudinal random effects model

Covariate	Reference category	OR	95% CI	*p*-value
Age	70–74 vs 65–69	0.968	0.873–1.074	0.538
	75–79 vs 65–69	0.901	0.793–1.025	0.113
	≥80 vs 65–69	0.685	0.589–0.797	<0.0001
Residential area	Rural vs Urban	1.728	1.576–1.895	<0.0001
House type	Apartment vs Detached	0.639	0.579–0.707	<0.0001
	Others vs Detached	0.617	0.541–0.704	<0.0001
Physical health	Good vs Poor	1.501	1.322–1.703	<0.0001
Mental health	Good vs Poor	2.062	1.874–2.269	<0.0001
Sex by Household composition^a^	female/couple vs male/couple	1.220	1.061–1.402	0.0052
	female/single vs male/single	1.873	1.473–2.383	<0.0001
	female/others vs male/others	1.122	0.969–1.299	0.121
	female/couple vs male/single	2.213	1.733–2.826	<0.0001
	female/single vs male/couple	1.033	0.903–1.180	0.638
	female/others vs female/single	0.849	0.745–0.967	0.014

^a^The model has a significant interaction; selected comparisons are presented

## Discussion

Our study aimed to determine factors that are significantly associated with the five domains of life satisfaction: health, economic, housing, family relations, and neighbor relations. Findings are consistent with some previous studies that indicate the importance of physical and mental health, financial strain, residential area, housing type, and living environment for LS among the older population. Our study found that physical and mental health were consistently significantly associated with satisfaction in each of these domains after adjusting for potential confounders. This finding aligns with many other studies [24, 28, 29, 31]. Many studies have also found that mental health symptoms such as depression, anxiety, and psychosomatic problems are associated with lower life satisfaction [25, 38, 46, 50]. However, our data only contained general mental health status information and did not provide specific mental health symptoms, clinical examination findings, chronic conditions, medication use, physical activities, or activities of daily living.

Living in a rural area and living with a spouse were associated with being satisfied with economic status, housing, family relations, and neighbor relations, but these factors were not connected to satisfaction with health. Living in a rural area may provide a relatively steady and close social network through regular contact over time, which provides support and satisfaction in multiple aspects of LS. This finding on residential area also supported the previous studies [56, 66]. Even though our study showed that living in a rural area was not associated with health satisfaction, other studies found higher levels of life satisfaction in urban elders than rural elders because of greater access to basic social and medical service [37, 46, 51].

Connections between physical environment, social environment, and life satisfaction was also observed in housing type and living arrangements. Compared to living in a detached house, living in an apartment was associated with satisfaction in economic status, housing, and family relationships, but a lack of satisfaction in neighbor relationships. Living alone appears to result in less frequent satisfaction than living with a spouse or in other household composition types, which is consistent with the previous studies [26, 37, 39–43]. It is known that living arrangements influence life satisfaction, as living alone increases anxiety around situations of sickness and financial difficulty. A study in China showed that those living in single-generation households had lower psychological well-being than those who living in three-generation households or skipped-generation households [52]. In our study, having enough financial resources provided significantly higher economic satisfaction and satisfaction with family relationships; however, this factor did not significantly affect satisfaction with health, neighbor relationships, or, specifically, housing. Nonetheless, having private health insurance, a factor associated with financial stability, was associated with housing satisfaction.

Some recent studies have shown that older age predicted an increase in life satisfaction [14, 15] but others suggested that life satisfaction peaked at the age of 65 and then decreased [12]. Others yet suggest that there is a very late, age related decline in life satisfaction in the oldest age groups [18, 19]. However, our study did not show any of these patterns. The results from the eight- year and nine-year longitudinal studies by Gana [67] and Rocke [68] showed that life satisfaction was rather stable. Similarly, our study also indicated that life satisfaction among individuals up to the age of 80 years remains relatively constant in terms of health and family relationships. This may be due in part to prolonged survival of those with genetic predisposition to good health and those with strong family supports who can report satisfaction in these areas into their more advanced years. In contrast, a rapid decline in satisfaction with the neighbor relationships for all ages was also seen.

Our findings provide valuable scientific evidence both for understanding why many studies have presented inconsistent results and for proposing that measuring one dimension of life satisfaction is not appropriate. Our study showed gender differences in satisfaction with health, economic status, family and neighbor relationships, but no difference in housing satisfaction. As expected, women were observed to be less often satisfied with their health than men, given that women tend to out-live men and subsequently experience more health-related problems and loneliness. This finding is consistent with other studies [23, 50]. Our study also showed that satisfaction in family and neighbor relationships were higher among women than men. Oshio [42] found gender differences in the associations of LS with family and social relations in Japan. For example, family relations were of more importance to men compared to women. In older men whose marital status remained stable, LS was also constant, while in case of women there was a decline in LS. In addition, LS in men increased with marriage while it had no significant role for women [42]. Social relationship is also a stronger determinant of life satisfaction in older women than in older men [42, 69]. Compared to old men, old women are more likely to use friends as their associates and give more support in order to maintain friendships, and maintain contact with extended family members as well as with friends [70, 71]. This may be attributable to women being more actively connected to family members, friends, and neighbors whereas most older men rely on their wives for social support and rewarding relationships [72] Another possibility is that more traditional patriarchal roles in the older population adversely affects mood in older married women, as suggested by Jang et al [73]. Women LS also increases with higher number of social activities and friend circle which is not that significant predictor of LS among the males [44].

A major strength of our study was the ability to examine changes in the multidimensional construct of life satisfaction over a period of six years using a large sample of longitudinal data. Most of the studies to date have been cross-sectional studies and have used a single measure of life satisfaction, which is questionable in validity. However, our study used multiple domains of life satisfaction, resulting in a more comprehensive assessment. The second strength of our work is the generalizability of the study results. As the findings are based on a nationally representative longitudinal sample, they can easily be generalized to the Korean older adults. A third strength is utilization of the financial stress index. For individual economic status, measures of income are often not precise and employing a valid measure of income is difficult. In our study, application of the financial stress index provided an accurate measurement of a subjects’ economic status and, to the best of our knowledge, no other study has used it. The fourth strength of our study is that we used the GEE modeling approach, which allows us to effectively deal with missing values and to take into account correlations between an individual’s repeated measurements.

This study also has several limitations. The variables in our study do not cover important potential factors related to the domains of life satisfaction such as activities of daily living. Another limitation is that the data does not contain health-related variables; additional medically-based health measures, including chronic disease, anxiety, depression, etc., would have further improved our understanding of life satisfaction in older adults. In addition, as indicated in many studies, social support and family support measures are additional important factors associated with life satisfaction in older adults. Unfortunately, such variables were not available for our study. In the GEE model, we assumed that data are missing at random. However, technically, it is not easy to show that this assumption is valid.

## Conclusion

While most studies have focused on overall life satisfaction, considering multidimensional life satisfaction is essential to gaining a complete picture. Our study showed that physical and mental health status was most significantly associated with a multidimensional construct of life satisfaction among the Korean older adults. Our study also showed that, depending on the domain, aging is negatively or positively related to life satisfaction. It indicates that a single domain of LS or overall LS will miss many important aspects of LS because age-related LS is multifaceted and complicated. Thus using a single dimension or simplified overall LS might not be appropriate for drawing conclusions when studying older adults. Further research including personal behaviors, social networks, and medical, psychological, and environmental variables needed to comprehensively understand, and subsequently improve, life satisfaction in the older population.

## Abbreviations

GEE: Generalized estimating equations
KreIS: Korean Retirement and Income Study
LS: Life satisfaction
